# Artificial snare technique for transfemoral repositioning of malpositioned central venous port catheter

**DOI:** 10.1186/s42155-025-00563-w

**Published:** 2025-08-22

**Authors:** Taha Yusuf Kuzan, Rüçhan Anbar

**Affiliations:** 1 Department of Radiology, Sancaktepe Şehit Prof. Dr. İlhan Varank Training and Research Hospital, University of Health Sciences, İstanbul, Türkiye; 2 Department of Thoracic Surgery, Sancaktepe Şehit Prof. Dr. İlhan Varank Training and Research Hospital, University of Health Sciences, İstanbul, Türkiye

**Keywords:** Central venous port, Port catheter, Malposition, Snare, Radiological intervention

## Abstract

Subcutaneously implanted port catheters are increasingly used in the management of long-term chemotherapy and provide great comfort to oncology patients. Although central venous ports are implanted with high technical success and low complication rates, catheter malposition may occur, resulting in port dysfunction. Various techniques exist for repositioning malpositioned catheters. This report describes the use of an artificial snare technique for the repositioning of a central venous port catheter that was identified as malpositioned six months following its insertion.

**Supplementary Information:**

The online version contains supplementary material available at 10.1186/s42155-025-00563-w.

## Background

Central venous ports are a commonly utilized approach for achieving central venous access, particularly for intravenous chemotherapy treatment of oncology patients. Compared to surgical implantations, port implantations guided by ultrasonography and fluoroscopy have been documented to demonstrate exceedingly high technical success and exceptionally low complication rates [1]. An important cause of central venous port catheter dysfunction is catheter malposition and migration, which is observed in approximately 1–2% of patients, depending on the location of the catheter and the technique used [2, 3]. Malposition may occur during or after catheter insertion. Various techniques have been described for repositioning the malpositioned catheter [4].

This study presents a case report of the repositioning of a port catheter using the artificial snare technique via percutaneous transfemoral access in a patient who exhibited port dysfunction due to catheter malposition six months following central venous port implantation.

### Main text

**Fig. 1:**
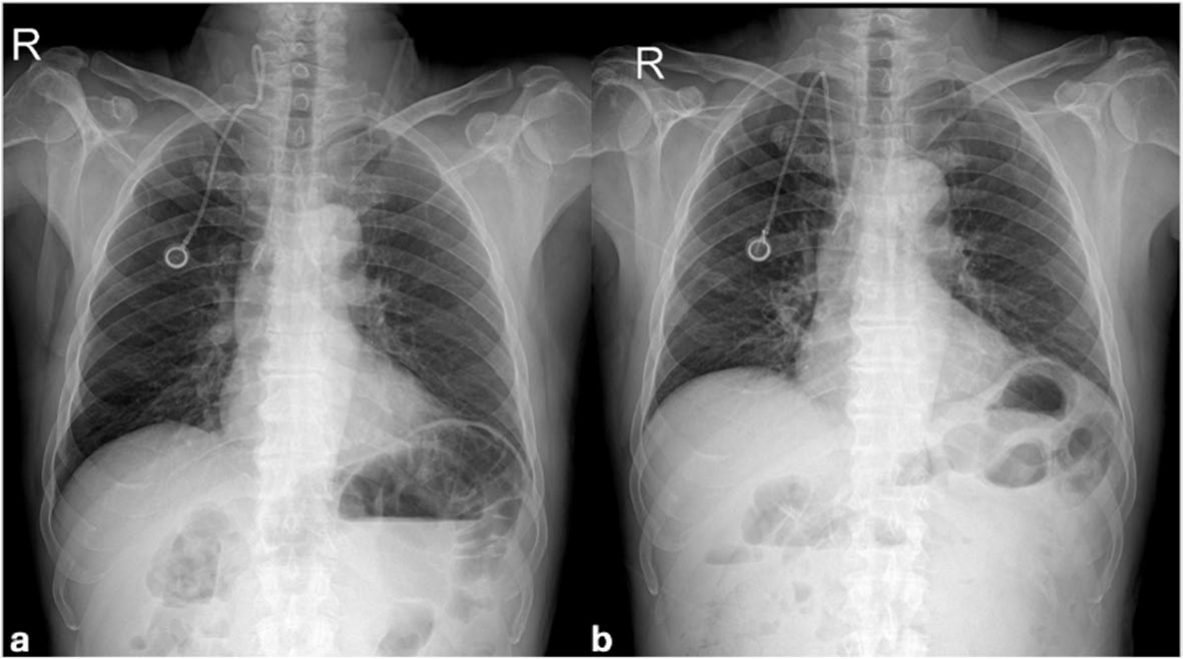
Chest radiography performed for port dysfunction showed that the port catheter was malpositioned in the right jugular vein (**a**, July 2024). The patient's last available radiological study showed that the catheter was in the superior vena cava (**b**, May 2024)

A 51-year-old male with a history of colon cancer was referred to interventional radiology due to an ineffective subcutaneous venous port that had been surgically inserted through the right jugular vein for chemotherapy six months prior. On imaging, it was determined that the tip of the port catheter was incorrectly positioned in relation to the right jugular vein (Fig. 1a, June 2024). Upon retrospective review of the most recent radiologic images of the port (Fig. 1b, May 2024), it was observed that the catheter tip was situated within the superior vena cava and that the port was functioning without any issues. Upon evaluation of the port’s position via fluoroscopy, it was observed that the catheter tip had curved from the entry site into the right internal jugular vein and had been displaced cranially within the vein. This finding led to the diagnosis of catheter migration, prompting the decision to pull the catheter tip back to its original position via the transfemoral route.

**Fig. 2:**
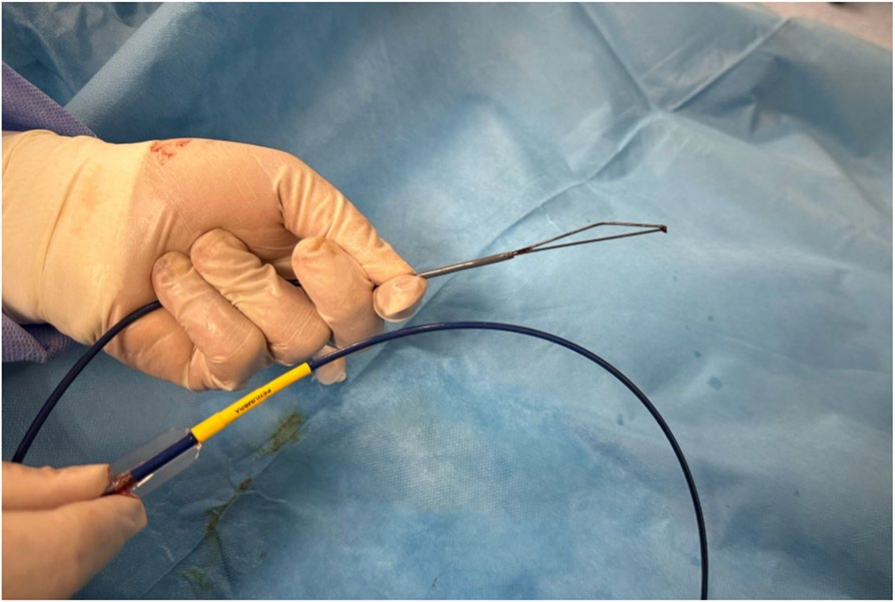
Artificial snare created using a long introducer and hydrophilic wire

The procedure was conducted under local anesthesia under ultrasound and fluoroscopy guidance. An 8 F valved sheath was inserted into the right common femoral vein using the Seldinger technique. Images obtained with the administration of contrast medium demonstrated that the catheter tip was situated within the right internal jugular vein and that the superior vena cava was patent. An artificial snare was created outside the patient using a 6 F 90 cm long introducer (088 Neuron Max, Penumbra, Alameda, California, USA) and a 0.035 inch × 260 cm hydrophilic guidewire (Glidewire, Terumo, Tokyo, Japan). To achieve this, the hydrophilic wire was gently folded in half and each end grasped at the distal end of the long introducer and pulled out from the proximal end. This constructed an artificial snare at the distal end of the long introducer, which could be manipulated with both ends of the hydrophilic wire exiting in a proximal position relative to the long introducer (Fig. 2). Utilizing an 8 F sheath positioned in the femoral vein, the right internal jugular vein was accessed with a long introducer equipped with an artificial snare at its distal end.

**Fig. 3:**
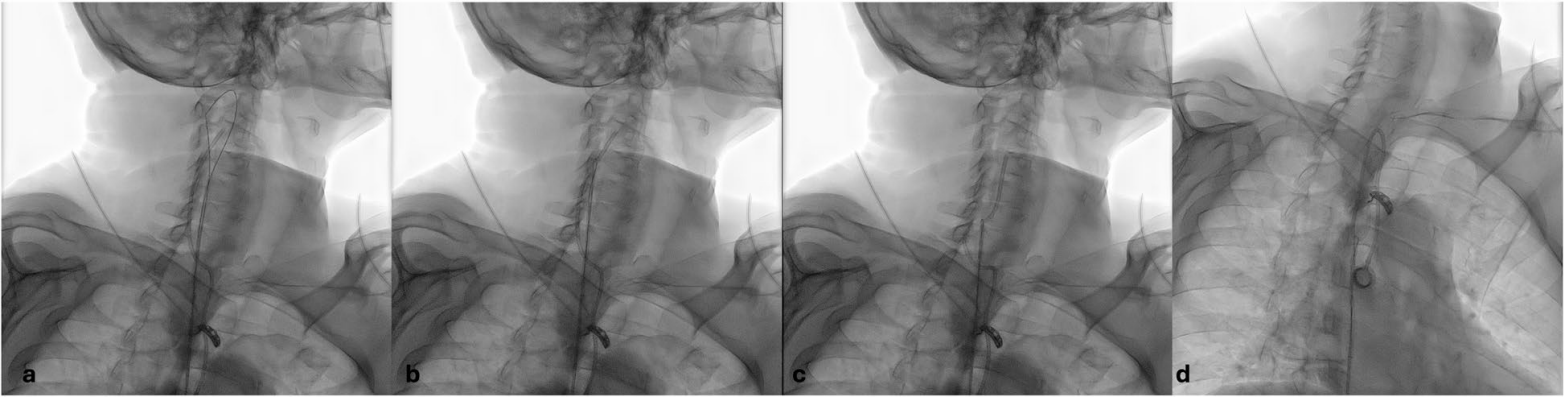
The process of repositioning a malpositioned port catheter with an artificial snare is described step by step. The wire, folded in half, was inserted through the long sheet to create an artificial snare (**a**). The tip of the catheter was then placed inside the artificial snare (**b**). The trapped catheter was then pulled downward (**c**), and the catheter tip was left in the superior vena cava (**d**)

The distal ends of the wire were then gently advanced and retracted, forming a loop that could expand and contract from the long introducer. Subsequently, both ends of the 0.035-inch guidewire were manipulated and pulled outward from the long introducer in a controlled manner, allowing for the gradual closure of the created snare until the port catheter was captured. During the course of the trials, the port catheter tip was positioned between the long introducer and the guide wire, and the catheter tip was grasped. Subsequently, the catheter tip was withdrawn and repositioned meticulously toward the cavoatrial junction (Fig. 3 and supplementary video 1).

**Fig. 4:**
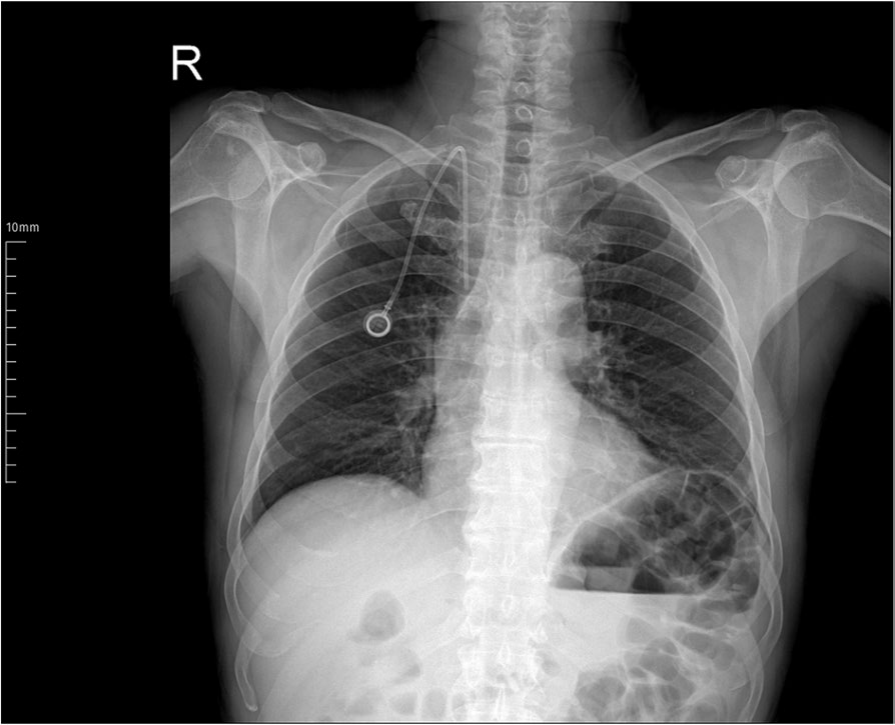
A chest radiograph obtained three months after the catheter was repositioned revealed that the catheter tip remained in the same location

The insertion of the Huber port needle was completed without incident, and contrast material was administered through the port to confirm catheter functionality. The patient's port was found to be actively functioning and the procedure was completed, although the angle created by the catheter in the jugular vein was acute and the distal end of the catheter terminated in a suboptimal position in the superior vena cava (Fig. 4). Following the procedure, the patient did not develop any new complications related to the port and chemotherapy was continued without any difficulties. The catheter was noted to be in the same position on the chest radiography obtained three months later.

In clinical practice, central venous port catheters are employed as a long-term, secure method for administering treatments such as parenteral nutrition, hematopoietic stem cell transplantation, and antibiotic infusion. In addition, they serve as reliable sources of permanent venous access for intravenous chemotherapy [1].

Migration represents one of the most well-documented complications associated with port catheters. The incidence of this phenomenon has been documented in various series at a rate of 0.9–1.8% [3]. The most direct method for addressing a malpositioned port catheter is to remove it and implant a new one. However, this approach carries inherent costs and potential complications, including pneumothorax, catheter reimplantation, or infection. Percutaneous transfemoral catheter repositioning is an efficacious procedure that is rapid, simple, and safe for both the patient and the practitioner. Furthermore, it has a low complication rate and a high technical success rate. The techniques of snare and angiography (Simmons or Pigtail) catheters are well-established methods for the repositioning of malpositioned central venous catheters. The choice of method for repositioning a port catheter following transfemoral access is dependent on the specific circumstances of the migration, including its location and the extent of its occurrence [5]. In this particular case, given that the catheter tip had migrated superiorly from the right jugular vein, the snare technique was deemed a more suitable approach. However, since a snare was not available, an "artificial snare" was created using a hydrophilic long guidewire and a long introducer. Although not identical, foreign body removal from the heart with a similar method has been reported in the literature [6]. This technique constitutes a cost-effective and efficacious option that can be used as an alternative method in cases where a snare is unavailable.

While catheter migration is predominantly observed during the initial post-procedural phase (≤ 30 days), instances of spontaneous malposition have also been documented in the late period. The precise mechanism of occurrence remains unclear. However, case reports have indicated that the extravascular component may be triggered by physical exertion, particularly in obese and large-breasted female patients. Conversely, the intravascular component may manifest in circumstances that elevate intra-abdominal pressure, such as coughing, sneezing, heavy lifting, and high-pressure drug infusion [3, 7]. Furthermore, the position of the port during the initial insertion is a significant factor in the potential for migration to occur.

Although the appropriate position of the catheter tip is still being debated, the cavoatrial junction area is considered ideal. In the optimal position, where the two vertebral body units below the inferior carina border are identified as the cavoatrial junction (CAJ), the catheter tip is located a few centimeters above and below the CAJ on anteroposterior chest radiographs. In the suboptimal position, the catheter tip was located in the superior vena cava (SVC) zone, which is situated below the designated upper margin of the SVC, or in the right atrium [8].

In this instance, the port placement was conducted via a surgical approach with ultrasound-guided puncture, without the utilization of fluoroscopy. The jugular vein puncture site was located at a superior level. The angle created by the port catheter at the entry point into the jugular vein was acute, and the port catheter tip remained suboptimally positioned within the superior vena cava.These factors were considered to contribute to the catheter migration observed in this case. For these reasons, the insertion of central venous port catheters under ultrasound and fluoroscopy guidance with a low puncture into the jugular vein, the insertion of the catheter through the jugular vein with a wide angle, and the leaving of the catheter tip near the atriocaval junction will reduce the risk of post-procedural migration.

## Conclusion

To conclude, interventional radiologists are involved in both the insertion of ports and the management of complications arising from port catheters, whether early or late. To prevent port catheter migration, venous access should be performed under ultrasound guidance and the catheter should be implanted in the appropriate position under fluoroscopic guidance. In the event of catheter dysfunction resulting from malposition, the catheter can be repositioned percutaneously via transfemoral route using a snare. Endovascular repositioning is a rapid, straightforward, and secure method with minimal complication rates and high technical success rates for both the patient and the practitioner. In instances where a snare is unavailable, the artificial snare catheter described in this case can be employed as an alternative approach for catheter repositioning.

## Supplementary Information

Supplementary Material 1.Video of percutaneous port catheter repositioning with artificial snare.

## Data Availability

All of the data that were generated or analyzed during this study are included in this article.
